# Supplementary material for: Democratizing AI: non-expert design of prediction tasks
Source: PeerJ Comput Sci. 2020 Sep 7;6:e296. doi: 10.7717/peerj-cs.296 (PMC7924542; doi:10.7717/peerj-cs.296)
Supplement: Supplemental Information 1 [file peerj-cs-06-296-s001.pdf]

Supplemental Materials for "Democratizing AI: Non-expert design of prediction tasks"

June 15, 2020

Contents

1 Task interfaces **1**

2 Table of proposed problems **7**

List of Figures

1 Problem proposal (phase one) task interface 3
2 Problem importance ranking and categorization (phase two) task interface—part 1 4
3 Problem importance ranking and categorization (phase two) task interface—part 2 5
4 Problem learnability (phase two) task interface 6
5 Data collection (phase three) task interface 6

1 Task interfaces

Here we detail the task interfaces workers used as part of the crowdsourcing (screenshots in Figs. 1–5).

The contents of the dropdown selection menus are not shown in the screenshots so we detail them here. The problem topics dropdown in Fig. 3 contains the options (top-to-bottom):

- "Health/Wellness"
- "Demographic (personal attributes)"
- "Politics or current events"
- "Factual (look up in encyclopedia)"
- "Other"
- "Unsure"

The learnability dropdown in Fig. 4 contains the options (top-to-bottom):

- "NO ONE will answer true"
- "FEW will answer true"
- "ABOUT HALF will answer true"

- “MOST will answer true”
- “EVERYONE will answer true”

Likert-scale dropdowns (used in the “Usefulness of input questions” in Fig. 3) contain the options (top-to-bottom):

- “Strongly disagree”
- “Disagree”
- “Neutral”
- “Agree”
- “Strongly agree”

Lastly, self-reported confidence dropdowns contain the options (top-to-bottom):

- “Very not confident”
- “Not confident”
- “Neutral”
- “Confident”
- “Very confident”

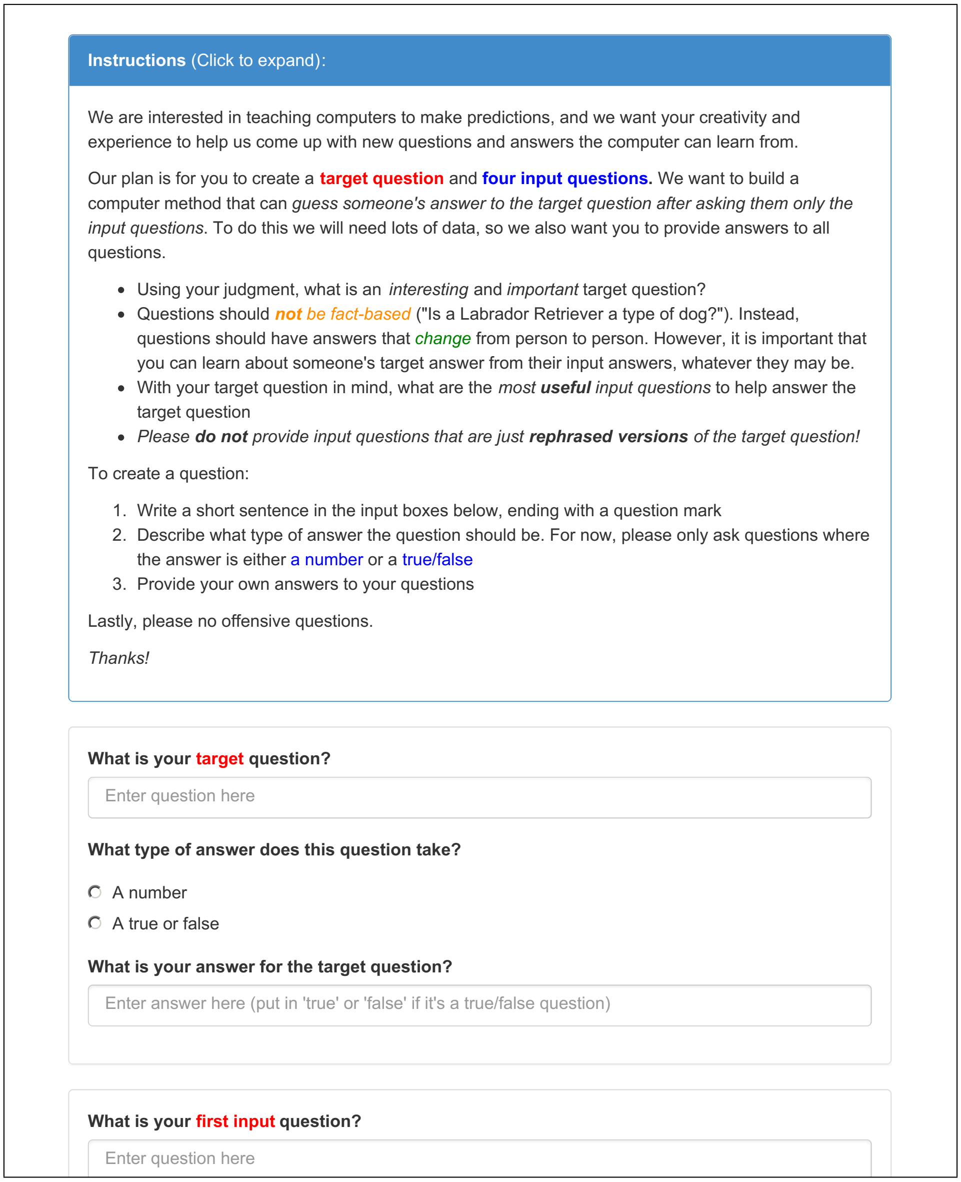

Instructions (Click to expand):

We are interested in teaching computers to make predictions, and we want your creativity and experience to help us come up with new questions and answers the computer can learn from.

Our plan is for you to create a **target question** and **four input questions.** We want to build a computer method that can *guess someone's answer to the target question after asking them only the input questions*. To do this we will need lots of data, so we also want you to provide answers to all questions.

- Using your judgment, what is an *interesting* and *important* target question?
- Questions should ***not*** *be fact-based* ("Is a Labrador Retriever a type of dog?"). Instead, questions should have answers that *change* from person to person. However, it is important that you can learn about someone's target answer from their input answers, whatever they may be.
- With your target question in mind, what are the *most* ***useful*** *input questions* to help answer the target question
- *Please* ***do not*** *provide input questions that are just* ***rephrased versions*** *of the target question!*

To create a question:

1. Write a short sentence in the input boxes below, ending with a question mark
2. Describe what type of answer the question should be. For now, please only ask questions where the answer is either a number or a true/false
3. Provide your own answers to your questions

Lastly, please no offensive questions.

Thanks!

What is your target question?

Enter question here

What type of answer does this question take?

- A number
- A true or false

What is your answer for the target question?

Enter answer here (put in 'true' or 'false' if it's a true/false question)

What is your first input question?

Enter question here

Figure 1: Problem proposal (phase one) task interface. The input question entry forms farther down the page are not entirely shown but they are identical to the target question entry form which is shown.

Comparing and classifying **problems**

Instructions

We have collected a number of **problems** and we want your help to categorize and assess their quality. Two problems are shown below.

- A problem consists of a **target question** and four **input questions**. Our goal is to guess someone's answer to the target question by only asking them the input questions. Answers to questions can be either numbers or true/false statements.
- We are especially interested in studying **important and useful** problems. Guessing that 1+1=2 may be less interesting than other problems, for example. Using your best judgment, which of the two problems below is **better** in this sense?
- We also want to know how **useful** the input questions are. Does it seem like answering the input questions will tell us about the target question, or do you think the questions are unrelated?
- Are any or all of the input questions **redundant** compared to the target question, meaning that they are essentially asking the same thing, just in a rephrased way?

To proceed, please read the two problems and then complete the following survey questions.

Thanks for your help!

Problems

First problem (placeholder)

	**Text of question**	**Type of answer**
Target question:	Here will be a question?	True/False
Input question:	Here will be another question?	Number
Input question:	Here will be another question?	True/False
Input question:	Here will be another question?	Number
Input question:	Here will be the last question?	True/False

Second problem (placeholder)

	**Text of question**	**Type of answer**
Target question:	Here will be a question?	True/False
Input question:	Here will be another question?	Number
Input question:	Here will be another question?	True/False
Input question:	Here will be another question?	Number
Input question:	Here will be the last question?	True/False

Figure 2: Problem importance ranking and categorization (phase two) task interface—part 1. This is the top half of the web interface; see Fig. 3 for the bottom half. In use, the web interface is a single scrollable page. When workers have accepted a real task, the instruction box at the top is presented initially collapsed, with a "click to expand" option made available in case the worker wants to reread the instructions.

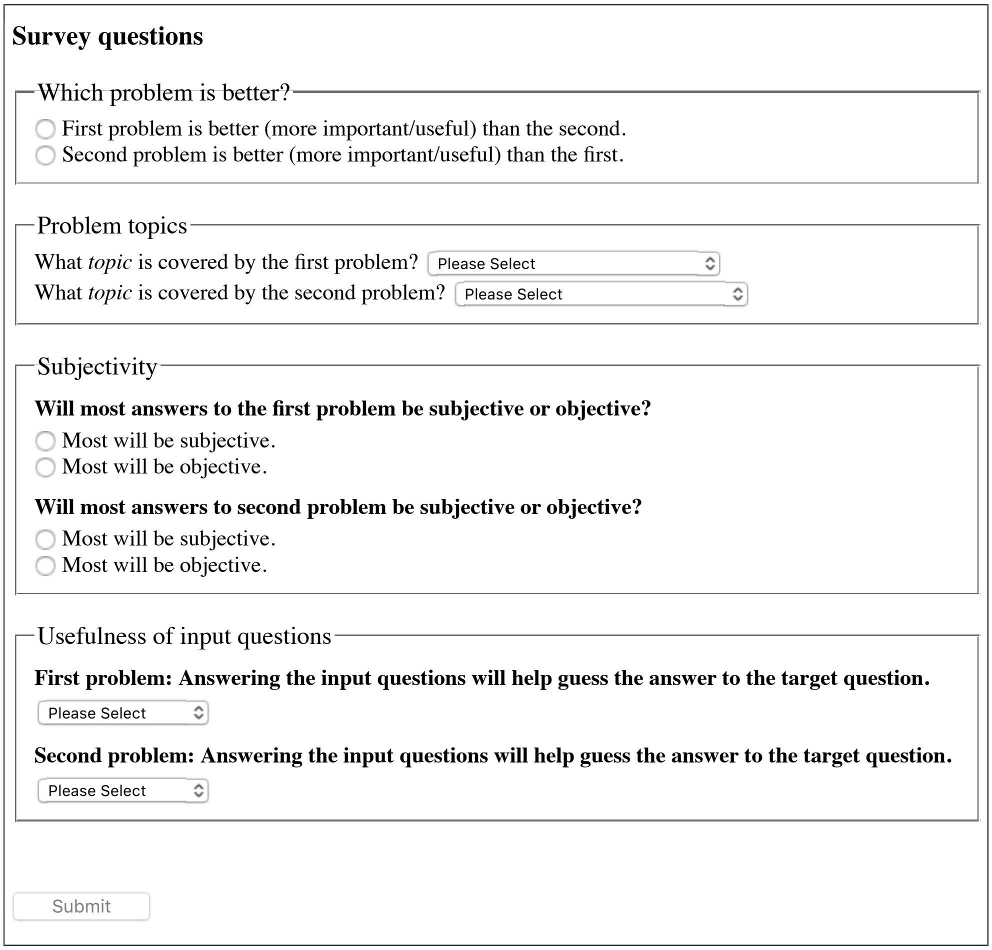
Survey questions

Which problem is better?

First problem is better (more important/useful) than the second.
Second problem is better (more important/useful) than the first.

Problem topics

What *topic* is covered by the first problem? Please Select
What *topic* is covered by the second problem? Please Select

Subjectivity

Will most answers to the first problem be subjective or objective?

Most will be subjective.
Most will be objective.

Will most answers to second problem be subjective or objective?

Most will be subjective.
Most will be objective.

Usefulness of input questions

First problem: Answering the input questions will help guess the answer to the target question.

Please Select

Second problem: Answering the input questions will help guess the answer to the target question.

Please Select

Submit

Figure 3: Problem importance ranking and categorization (phase two) task interface—part 2. This is the bottom half of the web interface; see Fig. 2 for the top half. In use, the web interface is a single scrollable page.

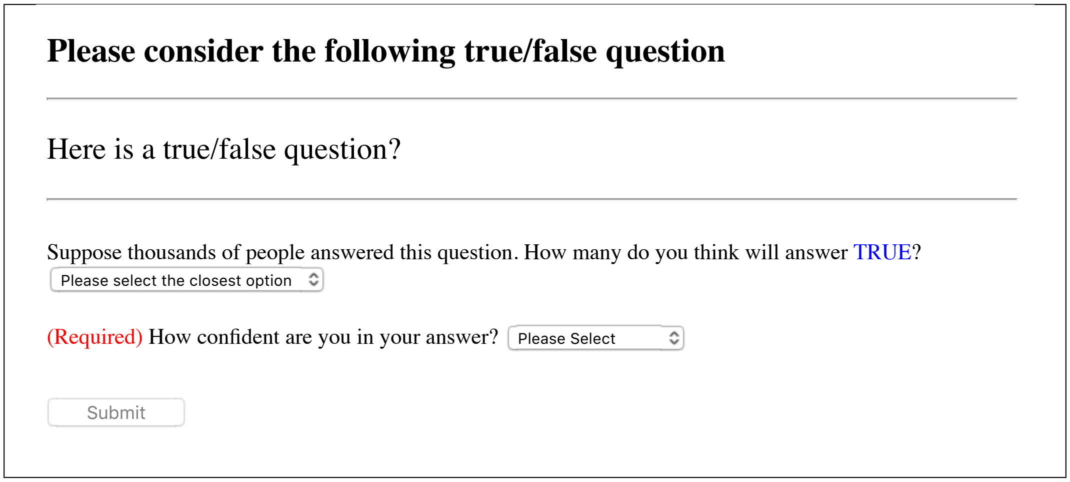

Figure 4: Problem learnability (phase two) task interface). For an actual prolem, the response fields for each question are dynamically generated as radio buttons (for true/false questions) or text entry fields (for numeric questions). The submit button is disabled until any/all radio buttons are checked and text fields contain numbers, as determined dynamically by javascript.

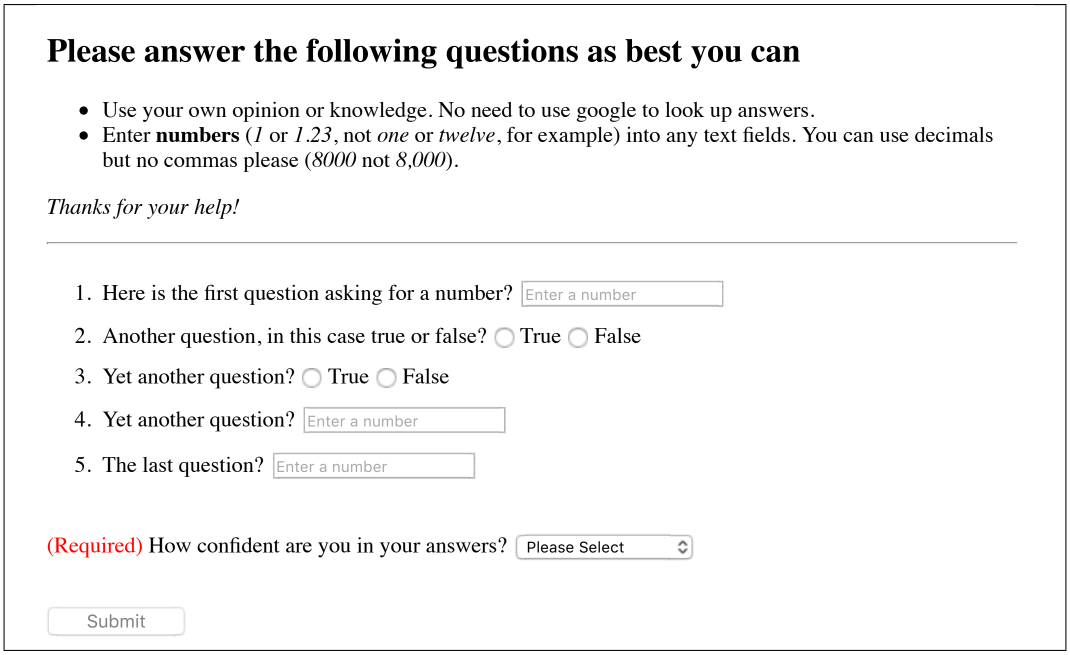

Figure 5: Data collection (phase three) task interface. For an actual problem, the response fields for each question are dynamically generated as radio buttons (for true/false questions) or text entry fields (for numeric questions). The submit button is disabled until any/all radio buttons are checked and text fields contain numbers, as determined dynamically by javascript.

2 Table of proposed problems

Workers proposed, categorized, and ranked $N = 50$ problems during our main experiment. Here we present all problems. Associated with each problem is its majority categorization along with the percentage of workers who gave that categorization. Problems are ranked from most "important and useful" (Rank 1) to least (Rank 50) according to worker judgment using an efficient spectral ranking algorithm based on pairwise comparisons of problems (*Which problem is better, A or B?*). See main text for details.

The rank 3 problem ("How far is the earth from the sun?") is the unitless regression problem where learning failed; see main text for discussion. As we progress down the ranking, we see that problems become less meaningful, demonstrating that the importance ranking can filter out at least the obviously unimportant problems.

Rank 1 (Politics/current events, 43.4%)

	Text of question	*Type of answer*
Target question:	What Category hurricane was Hurricane Harvey when it made landfall in Texas?	Number
Input question:	Are there 5 categories total for Hurricanes?	True/False
Input question:	Is it true that Hurricane Harvey was a weak hurricane?	True/False
Input question:	Is it true Hurricane Harvey was a category 4 with a peak wind speed of 131 mph before making landfall on Texas?	True/False
Input question:	Are categories of hurricanes determined by windspeed?	True/False

Rank 2 (Politics/current events, 94.8%)

	Text of question	*Type of answer*
Target question:	Has racial profiling in America gone too far?	True/False
Input question:	Do you feel authorities should use race when determining who to give scrutiny to?	True/False
Input question:	How many times have you been racially profiled?	Number
Input question:	Should laws be created to limit the use of racial profiling?	True/False
Input question:	How many close friends of a race other than yourself do you have?	Number

Rank 3 (Factual, 95.1%)

	Text of question	*Type of answer*
Target question:	How far is earth from the sun?	Number
Input question:	How big is the solar system?	Number
Input question:	How long does it take earth to circle the sun?	Number
Input question:	How strong is the sun's gravational pull on earth?	Number
Input question:	What is the strenght of earth's gravity?	Number

Rank 4 (Other, 53.7%)

	Text of question	*Type of answer*
Target question:	Is college a good investment?	True/False
Input question:	Is your degree in the STEM field?	True/False
Input question:	Are you able to get your degree without going into debt?	True/False
Input question:	Are you sure you know exactly what kind of job and what the pay is that you will be able to get with your degree?	True/False
Input question:	Have you considered other options?	True/False

Rank 5 (Politics/current events, 83.8%)

	Text of question	Type of answer
Target question:	is justice the same for everyone?	True/False
Input question:	should gay people be penalized	True/False
Input question:	is homosexuality a sin?	True/False
Input question:	is the justice system working well	True/False
Input question:	do women get equal treatment in the law	True/False

Rank 6 (Politics/current events, 97.4%)

	Text of question	*Type of answer*
Target question:	Do you think a wall should be built on the border with Mexico?	True/False
Input question:	Are you concerned about crime more from people of color than white people?	True/False
Input question:	Is immigration bad for the United States?	True/False
Input question:	Do feel at personal risk from immigrants?	True/False
Input question:	Do you watch Fox News?	True/False

Rank 7 (Demographic/personal, 87.1%)

	Text of question	*Type of answer*
Target question:	Will you go to college after high school?	True/False
Input question:	Do you want to make good money after high school?	True/False
Input question:	Do you want to have a good job after high school?	True/False
Input question:	Do you want to be successful after high school?	True/False
Input question:	How much money do you want to make after high school?	Number

Rank 8 (Demographic/personal, 81.7%)

	Text of question	*Type of answer*
Target question:	What is your annual income?	Number
Input question:	You have a job?	True/False
Input question:	How much do you make per hour?	Number
Input question:	How many hours do you work per week?	Number
Input question:	How many weeks per year do you work?	Number

Rank 9 (Politics/current events, 94.7%)

	Text of question	*Type of answer*
Target question:	Antifa is the most violent political group in America?	True/False
Input question:	Your political affiliation is republican?	True/False
Input question:	Violent is becoming common at protests?	True/False
Input question:	Anyone can use a vehicle as a weapon of mass murder?	True/False
Input question:	How many people were murdered in Charlottesville?	Number

Rank 10 (Health/Wellness, 96.1%)

	Text of question	*Type of answer*
Target question:	True or false, you lose more weight by staying to a strict diet and exercising well.	True/False
Input question:	True or false. Does running help with your health?	True/False
Input question:	How many calories should you limit yourself to if you're 5'8 and 240 lbs?	Number
Input question:	True or false. Is sugar good for your diet?	True/False
Input question:	True or false. Is an increase in fiber good for you?	True/False

Rank 11 (Health/Wellness, 91.2%)

	Text of question	Type of answer
Target question:	Do you have a good doctor?	True/False
Input question:	How many times have you had a physical in the last year?	Number
Input question:	How many times have you gone to the doctor in the past year?	Number
Input question:	How much do you weigh?	Number
Input question:	Do you have high blood pressure?	True/False

Rank 12 (Health/Wellness, 71.7%)

	Text of question	Type of answer
Target question:	Does the weather effect the way you feel?	True/False
Input question:	Do you feel sleepy on a cloudy day?	True/False
Input question:	When it is sunny and warm outside are you more energetic?	True/False
Input question:	Does the cold weather make you want to hibernate?	True/False
Input question:	Does the fall season depress you?	True/False

Rank 13 (Demographic/personal, 57.8%)

	Text of question	Type of answer
Target question:	Am I an introvert?	True/False
Input question:	Do I like social events?	True/False
Input question:	Do I get anxious easily?	True/False
Input question:	Do I hate teamwork?	True/False
Input question:	Do I cherish alone times?	True/False

Rank 14 (Other, 50.0%)

	Text of question	Type of answer
Target question:	I find social media to be educational for younger children?	True/False
Input question:	Social media should be accessed in school classrooms?	True/False
Input question:	Social Media helps inform children about things that tradional news does not show?	True/False
Input question:	Social media is helpful to further a child's intelligence?	True/False
Input question:	how many times do you let your child access social media per day?	Number

Rank 15 (Other, 57.3%)

	Text of question	Type of answer
Target question:	How many times should you allow your spouse to lie to you?	Number
Input question:	A person should disclose any extra-marital affairs to his/her spouse	True/False
Input question:	Infidelity is a deal-breaker in a marriage	True/False
Input question:	How many nights a week should your spouse spend hanging out with his/her own friends?	Number
Input question:	It is okay to marry someone who cheated on you before marriage	True/False

Rank 16 (Demographic/personal, 64.1%)

	Text of question	Type of answer
Target question:	Are children the most important thing in life?	True/False
Input question:	Do you have children?	True/False
Input question:	Do you work full time?	True/False
Input question:	How many children do you have?	Number
Input question:	Does your spare time revolve around your family?	True/False

Rank 17 (Health/Wellness, 88.2%)

	Text of question	*Type of answer*
Target question:	How many days a week do you exercise?	Number
Input question:	Do you exercise as part of a routine?	True/False
Input question:	Do you exercise on weekends?	True/False
Input question:	Do you exercise any weekdays?	True/False
Input question:	Do you take any weekdays off?	True/False

Rank 18 (Demographic/personal, 89.7%)

	Text of question	*Type of answer*
Target question:	Were you born to a highly educated family?	True/False
Input question:	Did your father graduate from college?	True/False
Input question:	Did your mother graduate from college?	True/False
Input question:	Did any of your siblings graduate from college?	True/False
Input question:	Did any of your extended family graduate from college?	True/False

Rank 19 (Politics/current events, 90.2%)

	Text of question	*Type of answer*
Target question:	Is it true or false that you approve of President Trump?	True/False
Input question:	Is it true or false that you voted for Donald Trump?	True/False
Input question:	Is it true or false that you are a republican?	True/False
Input question:	Is it true or false that you are a conservative?	True/False
Input question:	Is it true or false that you supported Hillary Clinton?	True/False

Rank 20 (Demographic/personal, 80.5%)

	Text of question	*Type of answer*
Target question:	Are you a college graduate?	True/False
Input question:	Do you go to college now?	True/False
Input question:	Did you go to college?	True/False
Input question:	Were you in a degree program?	True/False
Input question:	Did you finish your program?	True/False

Rank 21 (Demographic/personal, 56.9%)

	Text of question	*Type of answer*
Target question:	What is the best age to get married?	Number
Input question:	What is the best age to have your first child?	Number
Input question:	What is the best age to buy your first home?	Number
Input question:	What is the best age to start planning for job security?	Number
Input question:	What is the best age to start saving for retirement?	Number

Rank 22 (Health/Wellness, 61.3%)

	Text of question	*Type of answer*
Target question:	What is your weight in pounds?	Number
Input question:	What is your height in inches?	Number
Input question:	Are you male?	True/False
Input question:	Do you exercise regularly?	True/False
Input question:	How many meals a week do you eat meat?	Number

Rank 23 (Other, 69.3%)

	Text of question	*Type of answer*
Target question:	Is soccer a popular sport in the United States?	True/False
Input question:	Do a lot of people play soccer recreationally?	True/False
Input question:	Is it well regarded among the youth?	True/False
Input question:	Do people consider it an exciting sport?	True/False
Input question:	Do people prefer it over other sports?	True/False

Rank 24 (Demographic/personal, 50.0%)

	Text of question	*Type of answer*
Target question:	What age did your most memorable experience happen?	Number
Input question:	Did this happen when you were in college?	True/False
Input question:	Were any friends there?	True/False
Input question:	Was alcohol involved?	True/False
Input question:	How many other people were there?	Number

Rank 25 (Demographic/personal, 67.2%)

	Text of question	*Type of answer*
Target question:	How many vehicles are in your driveway?	Number
Input question:	How many vehicles belong to your family?	Number
Input question:	How many of your family's vehicles are kept at home?	Number
Input question:	Does your family park your vehicles in the home's driveway?	True/False
Input question:	How many of your family's vehicles are parked in the driveway?	Number

Rank 26 (Demographic/personal, 85.5%)

	Text of question	*Type of answer*
Target question:	Are you a college student?	True/False
Input question:	Do you live with your parents?	True/False
Input question:	Do you have a roommate?	True/False
Input question:	Do you have a college degree?	True/False
Input question:	Do you attend classes?	True/False

Rank 27 (Other, 59.3%)

	Text of question	*Type of answer*
Target question:	How many great pop acts have there been in the last 10 years?	Number
Input question:	Is Lady Gaga a great musician?	True/False
Input question:	How many great albums have been released in the last 10 years?	Number
Input question:	Is Madonna still relevant?	True/False
Input question:	Does pop music have a future?	True/False

Rank 28 (Factual, 45.5%)

	Text of question	*Type of answer*
Target question:	Is Mike Trout the best MLB player currently?	True/False
Input question:	What is the amount of RBI's that Mike Trout has in 2017 so far?	Number
Input question:	How many home-runs has Mike Trout hit in 2017 so far?	Number
Input question:	What is Mike Trout's batting average in 2017 so far?	Number
Input question:	What is Mike Trouts on base percentage in 2017 so far?	Number

Rank 29 (Demographic/personal, 91.5%)

	Text of question	*Type of answer*
Target question:	How old are you?	Number
Input question:	Do you have a degree?	True/False
Input question:	Are you retired?	True/False
Input question:	Are you married?	True/False
Input question:	Do you have a car	True/False

Rank 30 (Health/Wellness, 70.3%)

	Text of question	*Type of answer*
Target question:	Are you having a good day?	True/False
Input question:	Did any good occurrences happen within the course of today?	True/False
Input question:	Did the good occurrences of today outweigh the bad occurrences?	True/False
Input question:	Did you have a general feeling of happiness throughout the day?	True/False
Input question:	Did you feel overwhelmed with negative feelings (ex. sadness, irritability, guilt, etc.) throughout the day?	True/False

Rank 31 (Health/Wellness, 52.7%)

	Text of question	*Type of answer*
Target question:	Are you depressed?	True/False
Input question:	How many siblings do you have?	Number
Input question:	Did you graduate from college?	True/False
Input question:	How old are you?	Number
Input question:	Have you traveled overseas, out of the country?	True/False

Rank 32 (Demographic/personal, 90.3%)

	Text of question	*Type of answer*
Target question:	Are you married?	True/False
Input question:	You do not wear a ring on your left-hand ring finger?	True/False
Input question:	Do you live alone?	True/False
Input question:	How many incomes are reported on your tax return?	Number
Input question:	Do you celebrate a special anniversary every year?	True/False

Rank 33 (Factual, 61.7%)

	Text of question	*Type of answer*
Target question:	HOW MANY COUNTRIES ARE THERE IN THE WORLD?	Number
Input question:	ARE WE LIVING IN EARTH?	True/False
Input question:	IS HUMAN BEINGS HAVING SIXTH SENSE?	True/False
Input question:	IN A YEAR HOW MANY DAYS ARE THERE?	Number
Input question:	on which year second world war started?	Number

Rank 34 (Factual, 58.7%)

	Text of question	*Type of answer*
Target question:	Donald Trump is the 45th president.	True/False
Input question:	California is on the east coast.	True/False
Input question:	Earth is the _____ planet from the sun?	Number
Input question:	How many states are in the US?	Number
Input question:	How many is a dozen?	Number

Rank 35 (Demographic/personal, 93.4%)

	Text of question	*Type of answer*
Target question:	How old are you?	Number
Input question:	What year where you born?	Number
Input question:	What is the date today?	Number
Input question:	Have you had your birthday yet this year?	True/False
Input question:	How old will you be on your next birthday?	Number

Rank 36 (Other, 80.0%)

	Text of question	*Type of answer*
Target question:	Is Disneyworld is the best place for vacation?	True/False
Input question:	Is Disneyworld fun?	True/False
Input question:	Do you enjoy the attractions at Disneyworld?	True/False
Input question:	Does Disneyworld have the best live shows?	True/False
Input question:	Does Disneyworld have the best food?	True/False

Rank 37 (Factual, 95.6%)

	Text of question	*Type of answer*
Target question:	does the sun rise in the east?	True/False
Input question:	does the sun set in the west?	True/False
Input question:	is the sun pointing a direction?	True/False
Input question:	is the sun in the sky?	True/False
Input question:	does the earth rotate around the sun?	Number

Rank 38 (Demographic/personal, 54.5%)

	Text of question	*Type of answer*
Target question:	What size dress do you wear?	Number
Input question:	How much do you weigh in pounds?	Number
Input question:	What is your waist size in inches?	Number
Input question:	Do people consider you to be petite?	True/False
Input question:	Do people compliment you on your figure?	True/False

Rank 39 (Demographic/personal, 83.1%)

	Text of question	*Type of answer*
Target question:	When you were little did you ever go to kindergarden?	True/False
Input question:	Did you grow up in the northeast?	True/False
Input question:	Did you grow up in Pennsylvania?	True/False
Input question:	Did you go to kennett consolidated school district?	True/False
Input question:	Was your first school called ”the kindergarden” center?	True/False

Rank 40 (Demographic/personal, 46.7%)

	Text of question	*Type of answer*
Target question:	Are you human?	True/False
Input question:	Do you have an imagination?	True/False
Input question:	Do you have a sense of humor?	True/False
Input question:	Can you appreciate beauty?	True/False
Input question:	Have you ever had rebellious tendencies?	True/False

Rank 41 (Health/Wellness, 57.6%)

	Text of question	*Type of answer*
Target question:	You smoke marijuana?	True/False
Input question:	you like it?	True/False
Input question:	how many times have you smoked it?	Number
Input question:	at what age did you start smoking?	Number
Input question:	Marijuana affects your health negatively?	True/False

Rank 42 (Demographic/personal, 60.0%)

	Text of question	*Type of answer*
Target question:	Do you have a pet?	True/False
Input question:	Do you have a dog?	True/False
Input question:	Do you have a cat?	True/False
Input question:	Do you have a bird?	True/False
Input question:	Do you have a snake?	True/False

Rank 43 (Factual, 93.5%)

	Text of question	*Type of answer*
Target question:	Are there 30 days in August?	True/False
Input question:	How many months are there in the year that have 31 days?	Number
Input question:	are there 31 days in January and March?	True/False
Input question:	are there 31 days in december and october?	True/False
Input question:	Are there 31 days in may and july?	True/False

Rank 44 (Other, 76.1%)

	Text of question	*Type of answer*
Target question:	Pugs are nice pets?	True/False
Input question:	Pugs are good with kids?	True/False
Input question:	Pugs like to entertain people?	True/False
Input question:	Pugs are aggressive?	True/False
Input question:	Pugs are so ugly that they're cute?	True/False

Rank 45 (Other, 43.9%)

	Text of question	*Type of answer*
Target question:	are people are getting fatter all around the world?	True/False
Input question:	does everyone has a soul?	True/False
Input question:	is there is a heaven and a hell?	True/False
Input question:	are ghost real?	True/False
Input question:	is the locknes monster real?	True/False

Rank 46 (Factual, 76.9%)

	Text of question	*Type of answer*
Target question:	One plus one equal what number?	Number
Input question:	True or false that two plus two equal four?	True/False
Input question:	How many days in a week?	Number
Input question:	Is it true or false that pink is a color?	True/False
Input question:	Five plus Five equal what number?	Number

Rank 47 (Demographic/personal, 70.2%)

	Text of question	*Type of answer*
Target question:	Is Emilys hair brown?	True/False
Input question:	Emilys hair is dark?	True/False
Input question:	Emilys hair is blonde?	True/False
Input question:	Emilys hair is red?	True/False
Input question:	Emilys hair is black?	True/False

Rank 48 (Politics/current events, 52.6%)

	Text of question	*Type of answer*
Target question:	Is the president of the United States is Donald Trumph?	True/False
Input question:	Is the sky always Blue?	True/False
Input question:	What is 9 divided by 3?	Number
Input question:	What is 81 divided by 9?	Number
Input question:	What is 100 multiplied by 0?	Number

Rank 49 (Demographic/personal, 41.7%)

	Text of question	*Type of answer*
Target question:	How many weeks do you normally go between haircuts?	Number
Input question:	Your hair is less than shoulder length?	True/False
Input question:	You have bangs?	True/False
Input question:	Your hair grows fast?	True/False
Input question:	You have a stylist that you go to regularly?	True/False

Rank 50 (Factual, 64.4%)

	Text of question	*Type of answer*
Target question:	What date of the month is it?	Number
Input question:	What is 4+4?	Number
Input question:	Is tomorrow Wednesday?	True/False
Input question:	Does the sun rise in the west?	True/False
Input question:	How many days are in a week?	Number
